# Association between gut microbial change and acute gastrointestinal toxicity in patients with prostate cancer receiving definitive radiation therapy

**DOI:** 10.1002/cam4.6636

**Published:** 2023-11-03

**Authors:** Bum‐Sup Jang, Moon Gyu Chung, Dong Soo Lee

**Affiliations:** ^1^ Department of Radiation Oncology College of Medicine Seoul National University Seoul Korea; ^2^ Microbiome center Korea Research Institute of Bio‐medical Science Daejeon Korea; ^3^ Department of Radiation Oncology, College of Medicine The Catholic University of Korea Seoul Korea

**Keywords:** cancer, dysbiosis, microbiome, radiation therapy, toxicity

## Abstract

**Background:**

This prospective study investigated the association between gut microbial changes and acute gastrointestinal toxicities in prostate cancer patients receiving definitive radiation therapy (RT).

**Methods:**

Seventy‐nine fecal samples were analyzed. Stool samples were collected at the following timepoints: pre‐RT (prRT), 2 weeks after the start of RT (RT‐2w), 5 weeks after the start of RT (RT‐5w), 1 month after completion of RT (poRT‐1 m), and 3 months after completion of RT (poRT‐3 m). We computed the microbial community polarization index (MCPI) as an indicator of RT‐induced dysbiosis.

**Results:**

Patients experiencing toxicity had lower alpha diversity, especially at RT‐2w (*p* = 0.037) and RT‐5w (*p* = 0.003). Compared to patients without toxicity, the MCPI in those experiencing toxicities was significantly elevated (*p* = 0.019). In terms of predicted metabolic pathways, we found linearly decreasing pathways, including carbon fixation pathways in prokaryotes (*p* = 0.035) and the bacterial secretion system (*p* = 0.005), in patients who experienced toxicities.

**Conclusions:**

We showed RT‐induced dysbiosis among patients who experienced toxicities. Reduced diversity and elevated RT‐related MCPI could be helpfully used for developing individualized RT approaches.

## 1 INTRODUCTION

Radiation therapy (RT) is one of the most essential management options in prostate cancer.^1^, ^2^, ^3^ Moreover, recent advances in external beam RT technologies enabled more efficient eradication of tumors while minimizing treatment‐related detrimental effects.^4^, ^5^, ^6^, ^7^ Owing to the change in cancer epidemiologic trends, the 5‐year relative survival rates of prostate cancer have improved from 59.1% during 1993–1995 to 94.4% during 2015–2019. However, the incidence (3.1 from 1999 to 15.5 in 2019) and mortality (0.9 from 1999 to 1.5 in 2019) of prostate cancer have increased, according to the Korean nationwide cancer statistics reported in 2022.^8^ Although the beneficial and radical role of RT has been well demonstrated in prostate cancer, treatment‐related toxicities are major concerns due to their detrimental effect on patients' quality of life.^1^, ^9^, ^10^, ^11^, ^12^ Some patients can experience gastrointestinal (GI) toxicities, which may lead to treatment interruption or a decrease in patient quality of life.^11^, ^13^, ^14^

In the field of GI medicine, the gut microbiome is an emerging biomarker that is known to have a pivotal role in the development of diverse human diseases.^15^, ^16^ Several recent studies have described a significant connection between treatment outcomes or toxicities and the composition of the gut microbiome.^17^, ^18^, ^19^

We hypothesized that RT can potentially alter gut microbial composition or diversity and sought to unveil the relationship between gut microbial changes and acute GI toxicities in over a period of time. Because relatively high RT doses are administered in definitive treatment of prostate cancer compared to other pelvic malignancies, we also hypothesized that we could identify toxicity‐related microbial features in the current study.

## 2 METHODS

### 2.1 Study design and patient eligibility

We obtained written informed consent from each patient before study enrollment. The eligibility criteria were as follows: patients who were aged ≥20 years and provided informed consent; patients who were pathologically confirmed with pelvic malignancy before study entry; and patients who were planned to undergo definitive RT. Patients were excluded from the study with the following conditions: patients who had evidence of distant metastasis; patients who were concomitantly diagnosed with other untreated primary tumors; patients who did not complete the planned RT course; patients who had a previous receipt history of pelvic RT; and patients who refused additional fecal sampling during and after treatment (RT). According to the study protocol, stool samples were collected at designated timepoints as follows: pre‐RT (prRT) (1st), 2 weeks after the start of RT (RT‐2w) (2nd), 5 weeks after the start of RT (RT‐5w) (3rd), 1 month later from the end of RT (poRT‐1 m) (4th), and 3 months later from the end of RT (poRT‐3 m) (5th). Between August 2019 and June 2021, a total of 17 patients were enrolled in the study. All registered patients were male and were diagnosed with prostate cancer. Among these patients, one patient refused to continue further study involvement after the first sampling and was excluded from the study. Finally, 16 patients with 79 fecal samples (excluding one lost sample) were collected and analyzed for this study.

### 2.2 Treatments

All registered study population underwent curative RT. RT was performed using two arcs of the volumetric modulated arc therapy (VMAT) technique with 10 megavoltage photons. The Roach equations were applied to estimate seminal vesicle (SV) and pelvic lymph node (LN) involvement and criteria of approximately ≥20% were considered as potential risk of involvement. Target volumes and dose prescriptions (total and fractional doses) were decided according to the National Comprehensive Cancer Network (NCCN) prostate cancer risk classification and Roach equations. RT fields were deemed local fields if the patients received RT to the prostate or prostate/SV and large fields if the patients received RT to the pelvic LN stations along with the prostate/SV. Total RT doses were[PTV]1) and 45–50.4 Gy at 1.8 Gy per fraction in the low‐dose area (PTV2). Treatment characteristics are summarized in Table 1. Antihormonal treatment was administered as neoadjuvant therapy in 12 (75%) patients, and therapy was maintained thereafter during the entire sampling period.

**TABLE 1:** Baseline patient, tumor and treatment characteristics (N = 16).

Characteristics		N (%)
Age (year)		
Mean ± SD		73.1 ± 7
cT‐stage	cT1	4 (25)
	cT2	6 (37.5)
	cT3a	1 (6.3)
	cT3b	4 (25)
	cT4	1 (6.3)
cN‐stage	cN0	12 (75)
	cN1	4 (25)
Total Gleason Score	6	5 (31.3)
	7	9 (56.3)
	8	2 (12.5)
Initial PSA (ng/mL)		
Mean ± SD		49.1 ± 115.3
Number of total biopsy core		
Median (range)		12 (8–20)
Biopsy cancer percentage (%)		
Mean ± SD		44.5 ± 27.2
RT volume	Prostate	6 (37.5)
	Prostate/SV	2 (12.5)
	Prostate/SV/Pelvic LNs	8 (50)
Total RT dose (high‐dose area) (Gy)	69.6	3 (18.8)
	72	12 (75)
	74.4	1 (6.3)
Total RT dose (low‐dose area) (Gy)	0	8 (50)
	45	5 (31.3)
	50.4	2 (12.5)
	54	1 (6.3)
Use of antihormonal treatment	No	4 (25)
	Yes	12 (75)

Abbreviations: PSA, prostate specific antigen; RT, radiation therapy.

### 2.3 Restriction of diet and medication

Because dietary habits and medications can lead to potential shifts in microbiome abundance and distribution, we instructed that they should maintain the usual dietary pattern. We examined all medication histories of patients and instructed them that maintenance of preexisting medication due to chronic illnesses is allowed but intake of additional new drugs such as antibiotics, antacids, or probiotics is not recommended during sampling periods if possible.

### 2.4 Toxicity assessment and follow‐up

Patient history taking, physical examination, and toxicity evaluation were routinely performed at each follow‐up. Bowel and urinary toxicity were recorded based on patient‐reported toxicity outcomes every week during RT. After RT, patient follow‐up was conducted at 2 weeks, 5 weeks, 3 months, 6 months, and every 6 months thereafter or more frequently upon clinical needs. Bowel toxicity was mainly compared between patients with toxicity and those without toxicity. Toxicity was regarded as positive (+) when bowel habit change was prominent compared with baseline (≥ grade 1 acute lower gastrointestinal toxicity by Radiation Therapy Oncology Group Common Toxicity Criteria). Laboratory tests (including prostate‐specific antigen [PSA]) and imaging studies were performed when clinically indicated.

### 2.5 Sample collection, DNA extraction, and 16S rRNA gene amplicon sequencing

Fecal samples were collected from individual patients in autoclaved tubes and stored at −80°C. Bacterial genomic DNA from the fecal samples was extracted and quantified by the QIAamp DNA Stool Mini Kit (Qiagen, Hilden, Germany) according to the manufacturer's instructions for next‐generation sequencing (NGS) analysis. NGS library sequencing was performed following the manufacturer's Illumina iSeq library preparation protocol provided by Illumina (San Diego, CA, USA). Partial 16S rRNA gene sequences were amplified from extracted DNA using the StepOne Plus Real‐Time PCR System (Applied Biosystems, Foster City, CA, USA), which targets the V4 region of the 16S rRNA gene sequence. The amplicons were purified using AMPure beads to remove unused primers. An additional 8 cycles of PCR were performed using Illumina barcoded adapters to prepare the sequencing libraries. Mixed amplicons were pooled, and sequencing was performed with the Illumina iSeq100 sequencing system (Illumina, San Diego, California, USA). Classification and identification of 16S rRNA gene sequences for phylogenetic analysis and taxonomic assignment of the quality‐filtered sequences were processed in the Microbiome Taxonomic Profiling (MTP) pipeline of EzBioCloud (https://help.ezbiocloud.net/ezbiocloud‐apps/).

### 2.6 Statistical analysis and bioinformatics

The Shannon index was compared at each timepoint using the Wilcoxon rank‐sum test. Beta diversity distance was measured by principal coordinates analysis (PCA) based on generalized UniFrac^20^ from the species level of taxa, including unclassified OTUs. Data were normalized to the maximal read count. To identify beta set significance, we performed permutational multivariate analysis of variance (PERMANOVA) in a pairwise manner. The number of permutations was 999, and interset *p* values were estimated.

To find differential microbiota between groups, we used the publicly available toolbox “Statistical Inference of Associations between Microbial Communities And host phenoTypes”^21^ (SIAMCAT, https://github.com/zellerlab/siamcat). SIAMCAT provides an analysis pipeline for preprocessing, association testing, and statistical modeling of microbiome data. In the current study, we performed association testing between groups using SIAMCAT, which provides the abundances of the differential features. Features were filtered based on abundance using a cutoff of 0.01. The statistical significance was calculated by a Wilcoxon test using the Benjamini–Hochberg multiple correction method.

We computed the microbial community polarization index (MCPI), which was adopted from a previous Nakatsu et al. study,^22^ as an indicator of RT‐induced dysbiosis. Based on the association test, differential microbial taxa were selected as representative microbiota for each group. Then, the MCPI of the sample j was computed as follows:

$$\;{\text{MCPI}}_{j}=\ln\left[\frac{\left(\Pi A_{\mathrm{ij}}\;\text{in}\;T\right)}{\left(\Pi A_{\mathrm{ij}}\;\text{in}\;N\right)+\varepsilon }+1\right]$$

where $A_{\mathrm{ij}}$ is the abundance of microbial taxa i in toxicity, and no toxicity groups denoted as T, and N, respectively. MCPI was compared using analysis of variance (ANOVA).

A linear mixed effect (LME) model was adopted in the current study to evaluate time effects after RT with respect to microbial features within subjects. In terms of toxicity, the model was fitted by an interaction term with quadratic days after RT x field, the use of antihormone therapy, the event of toxicity, and age. In terms of the RT field, the interaction term with quadratic days after RT x toxicity was used instead. The dependent variable was the abundance of a microbiota, and the random‐effect variable was the subject number. After LME models were fitted, predictive margins and contrast of predictive margins between groups were calculated and plotted. The significance with respect to the contrast of predictive margins was estimated by the chi‐squared test. LME model establishment and Wilcoxon rank‐sum tests were performed by using STATA 17 (StataCorp LLC, TX, USA).

Functional biomarker discovery was performed by the Kruskal–Wallis H test to find differential Kyoto Encyclopedia of Genes and Genomes (KEGG) orthology according to timepoints. Based on orthology with a raw *p* value <0.05, Minpath^23^ and PICRUSt^24^ tools were used to infer metabolic pathway abundances. Significant pathways with raw *p* value <0.05 were collected, and their predicted abundances were plotted. This comparative functional biomarker analysis was performed within the EzBioCloud Apps platform (https://help.ezbiocloud.net/ezbiocloud‐apps/).

## 3 RESULTS

Among seven patients who experienced acute GI toxicities, six were in grade 1 and one was grade 2 (9 days treatment interruption due to toxicity). Three patients experienced persistent acute GI symptoms until RT completion, and symptoms were subsided in four patients. Among seven patients, five received large field RT and the remaining two received local field RT. As shown in Table S1, we found that all patients experiencing toxicity recovered 4‐week after RT. With median follow‐up of 298.5 days (range, 107–567), PSA levels after treatment remained consistently low, with all patients achieving PSA nadir values of median 0.01 ng/mL (range, 0.01–2.51).

### 3.1 Alpha and beta diversity according to toxicity

**FIGURE 1:**
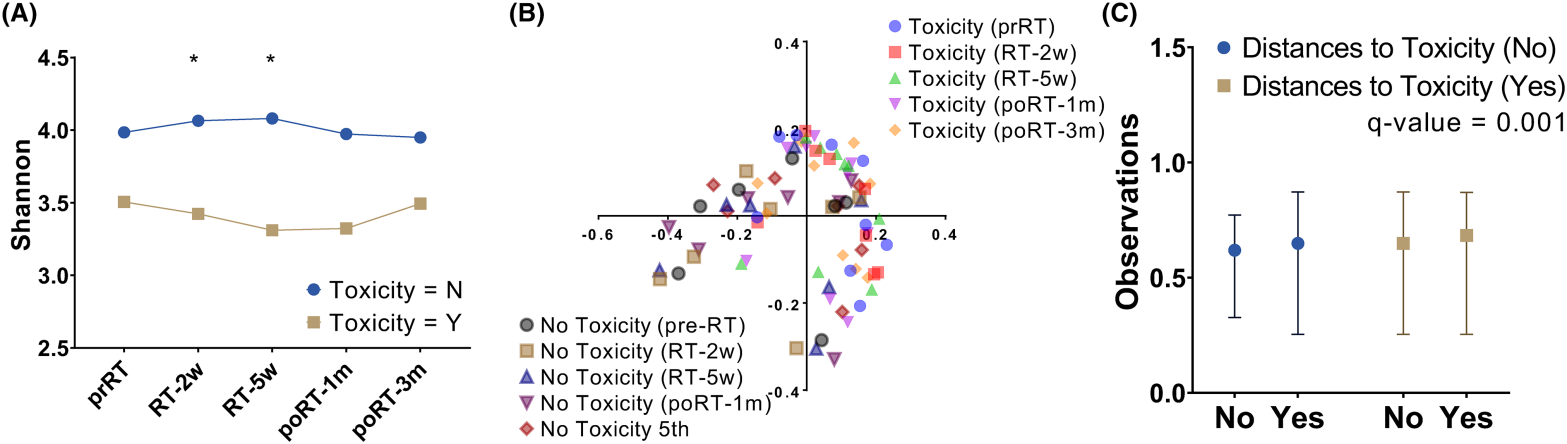
According to the presence of toxicity, alpha diversity (A), beta‐diversity distance (B), and PERMANOVA results (C) are plotted. RT, radiation therapy; PERMANOVA, permutational multivariate analysis of variance; prRT, pre‐RT; RT‐2w, 2 weeks after the start of RT; RT‐5w, 5 weeks after the start of RT; poRT‐1 m, 1 month after the end of RT; poRT‐3 m, 3 months after the end of RT. The *Q*‐value was estimated by the Benjamini–Hochberg multiple correction method.

A total of seven patients experienced ≥ grade 1 acute GI toxicities. In terms of alpha diversity, we found different patterns in the change in the Shannon index between patients showing toxicity and no toxicity. Overall, patients experiencing toxicity demonstrated lower diversity than those having no toxicity (Figure 1A). In time series samples, we found two time points reflecting significant differences between patients experiencing toxicity and no toxicity: RT‐2w (*p* = 0.037) and RT‐5wk (*p* = 0.003), indicating the manifested difference in alpha diversity in the middle of RT. In terms of beta diversity, we plotted the beta diversity distance according to the timepoints and the presence of toxicity (Figure 1B), demonstrating that patients experiencing toxicity had a less variable range of beta diversity than those without toxicity across timepoints. Figure 1C shows the result of PERMANOVA for beta‐diversity distance, resulting in a significant difference (*q*‐value = 0.001). However, we did not find any significant difference in beta‐diversity distance across timepoints within patients with toxicity and those without toxicity (all *q*‐value = 1.000, Figure S1).

### 3.2 Differential microbiota and the time‐dependent change in microbiota

**FIGURE 2:**
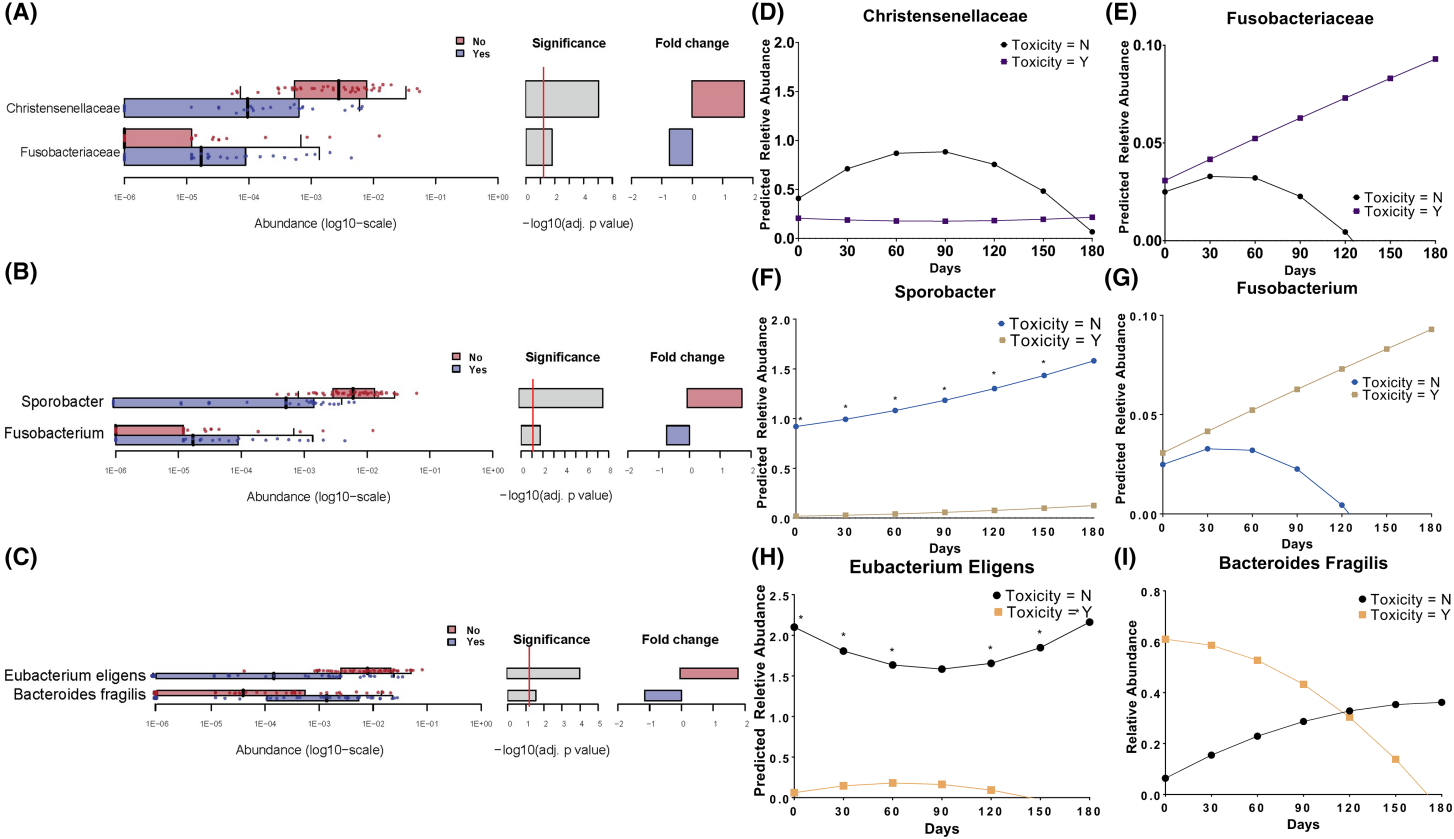
Differential taxa according to the presence of toxicity at the family level (A), genus level (B), and species level (C). For each differential taxa, a linear mixed effect model was fitted, and prediction plots are shown at the family level (D, E), genus level (F, G), and species level (H, I). Asterix represents that *p* value was less than 0.05.

Differential microbiota at the family, genus, and species levels in terms of toxicity were explored. Full significant associated features in terms of toxicity are provided in Figure S2A–C. Of those, a microbiota showing the largest generalized fold change toward each group was selected and tested within a linear mixed effect model. *Christensenellaceae* and *Fusobacteria* (family level, Figure 2A), *Sporobacter* and *Fusobaterium* (genus level, Figure 2B), and *Eubacterium eligens* and *Bacteroides fragilis* (species level, Figure 2C) were significantly enriched in patients showing no toxicity and toxicity, respectively. LME analysis demonstrated that *Christensenella* abundance showed a trend of first increasing and then decreasing over the first 90 days after RT in patients without toxicity (Figure 2D). *Fusobacteriaceae* (Figure 2E) and *Fusobacterum* (Figure 2G) demonstrated similar abundance trends, showing higher abundances in patients experiencing toxicity than in those without toxicity. The abundance of *Sporobacter* (Figure 2F) was consistently and significantly increased across timepoints in patients without toxicity. At the species level, *Eubacterium eligens* showed a U‐shaped abundance pattern from the start of RT in patients without toxicity (Figure 2H). Except at 90 days after RT, this trend was significant at all timepoints. Although *Bacteroides fragilis* showed the opposite trend of abundance between patients with and without toxicity (Figure 2I), the trend was not statistically significant.

### 3.3 The change in MCPI across timepoints

**FIGURE 3:**
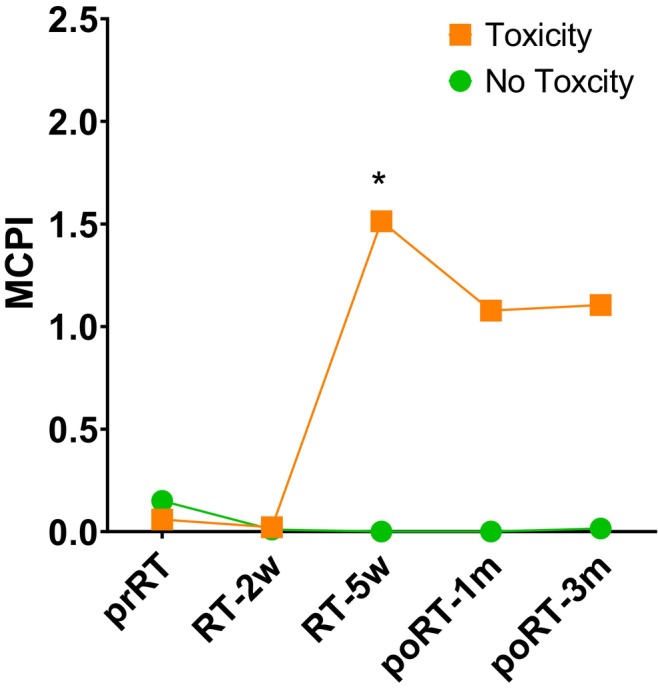
The change in RT‐related MCPI based on the family level of taxa, according to the presence of toxicity. Asterix represents that *p* value was less than 0.05. RT, radiation therapy; MCPI, microbial community polarization index; prRT, pre‐RT; RT‐2w, 2 weeks after the start of RT; RT‐5w, 5 weeks after the start of RT; poRT‐1 m, 1 month after the end of RT; poRT‐3 m, 3 months after the end of RT.

Based on differential microbial taxa between patients experiencing toxicity and those experience no toxicity (Figure 2A–C), we computed the sum product of abundance of differential taxa in each taxonomic. Thus, sum product of abundance of *Fusobacteriaceae*, *Fusobacterim*, *Bacteroides fragilis* and sum product of abundance of *Christensenellaceae*, *Sporobacter*, *Eeubacterium eligen* were calculated. Then, their log‐ratio was computed and compared between two groups (Figure 3). In patients who experienced toxicity, we observed the elevation of MCPI in the last part of RT session and slight decrease until 3 months after the completion of RT. Meanwhile, low level of MCPI was maintained during RT and after RT in patients without toxicity. Between two groups, significant difference in MCPI was identified at the time of 5 weeks after the start of RT (ANOVA *p* = 0.019).

### 3.4 Trend of functional pathways according to timepoints

**FIGURE 4:**
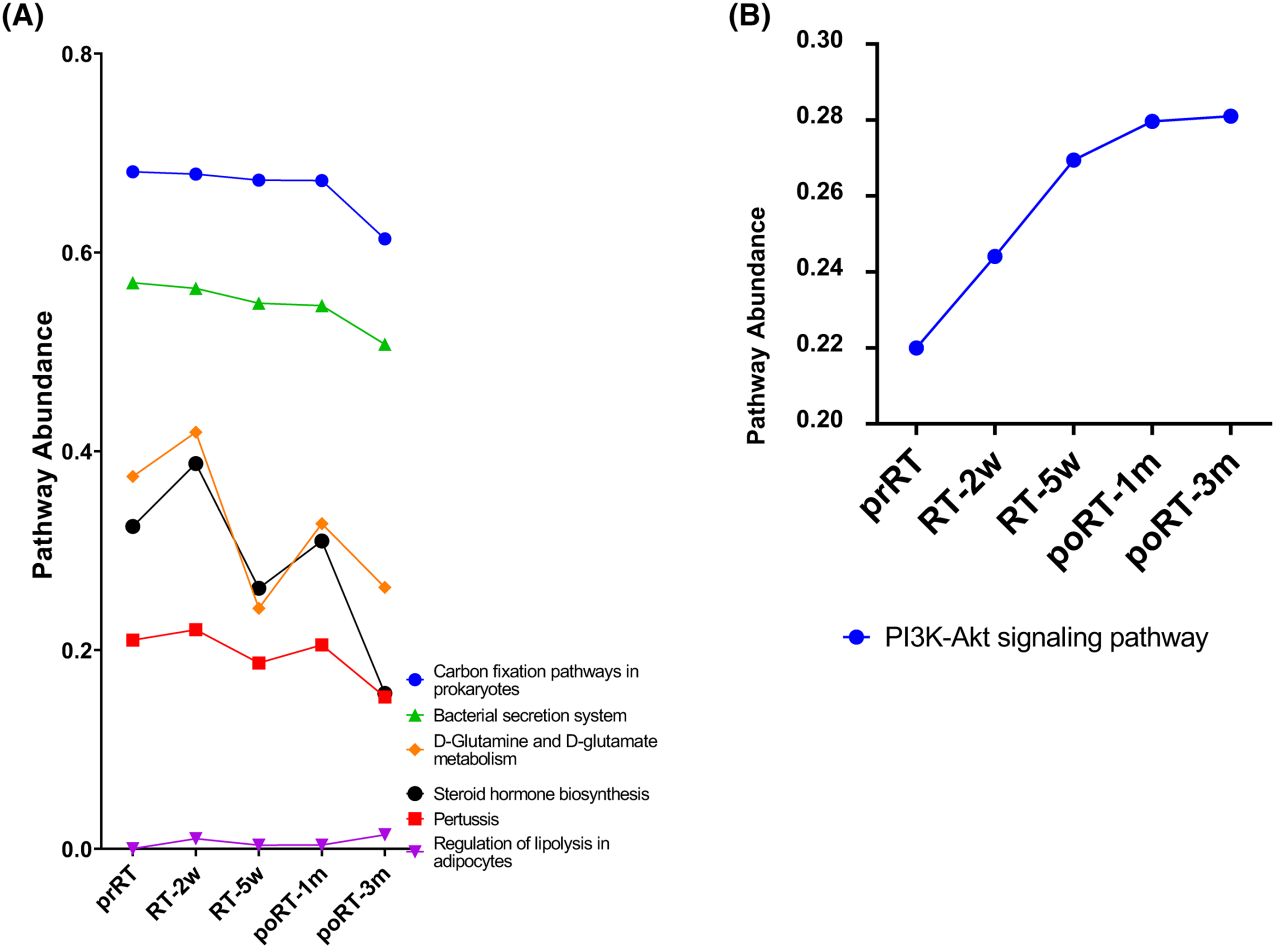
The change in functional pathways according to timepoints in patients who experienced toxicity (A) and no toxicity (B). RT, radiation therapy; prRT, pre‐RT; RT‐2w, 2 weeks after the start of RT; RT‐5w, 5 weeks after the start of RT; poRT‐1 m, 1 month after the end of RT; poRT‐3 m, 3 months after the end of RT.

We explored the functional pathways in groups stratified by the presence of toxicity. In patients who experienced toxicity (Figure 4A), we found linearly decreasing pathways, including carbon fixation pathways in prokaryotes (*p* = 0.035) and bacterial secretion systems (*p* = 0.005). Additionally, we found several increasing‐decreasing trends in metabolic pathways, including pertussis, D‐glutamine and D‐glutamate metabolism, and steroid hormone biosynthesis. In patients who did not experience toxicity, the PI3K‐Akt signaling pathway showed an increasing trend during RT and a saturated trend after RT (Figure 4B). Detailed predicted pathway abundances are summarized in Table S2.

## 4 DISCUSSION

We demonstrated the impact of RT on the patient's gut microbiome in a time‐dependent manner in terms of diversity and MCPI. The MCPI developed in the current study seemed to represent the dynamics of the microbiome in prostate cancer patients receiving RT. Additionally, nearly 1 month after the completion of RT, some functional pathways recovered to their pre‐RT levels.

Gastrointestinal (GI) toxicities of pelvic RT are associated with reduced diversity and dysbiosis within the gut.^19^ Accumulating studies^25^, ^26^, ^27^ have shown that decreased alpha diversity is associated with the status of inflammatory bowel disease. In line with this, reduced diversity can be related to the inflammatory status induced by RT. In fecal samples from 18 cervical cancer patients, Wang et al.^28^ demonstrated that enteritis was characterized by significantly reduced alpha diversity with a relatively lower abundance of *Bacteroides*. However, it should be noted that fecal samples were obtained before and the first day after RT. Meanwhile, in the current study, we analyzed more serial samples by collecting them before, during, and after RT. Our results showed that alpha diversity was significantly decreased at 2 and 5 weeks after RT in patients experiencing toxicity compared with those experiencing no toxicity. Based on this finding, we hypothesize that the diversity disturbance induced by RT could be initiated in the middle of the RT session.

In addition to reduced diversity, another dysbiosis within the gut can be induced by RT. In 35 patients with a new diagnosis of locally advanced cervical cancer, Mitra et al.^29^ analyzed the association between the Expanded Prostate Cancer Index Composite (EPIC) instrument and the gut microbiome. The EPIC questionnaire aims to measure patient‐reported toxicity to evaluate bowel and urinary toxicity. Relatively higher abundances of *Phascolarctobacterium*, *Lachnospiraceae*, *Veillonella*, *Erysipelotrichaceae*, and *Faecalitalea* were found in patients experiencing high toxicity. The LefSe analysis revealed that *Sutterella*, *Finegoldia*, and *Peptococcaceae (Clostridia)* demonstrated the highest differential abundance in patients who reported high toxicity. In prostate cancer, Ferreira et al.^30^ collected stool samples (*N* = 32) from pre‐RT up to 12 months post‐RT to assess early enterotoxicity induced by RT. Longitudinal analysis showed that RT‐induced enteropathy was associated with higher abundances of microbes, including *Roseburia*, *Clostridium IV*, and *Faecalibacterium*. Similarly, we performed longitudinal analysis at the family, genus, and species levels and found that *Sporobacter* abundance, which is known to be enriched in healthy people, significantly increased during and after RT for patients without toxicity compared to patients with Crohn's disease.^31^ In addition, RT caused a dynamic change in *Eubacterium eligens abundance—*abundance decreased during RT and recovered to baseline after the completion of RT—in patients without toxicity. A previous study^32^ analyzing fecal samples from nine gynecologic cancer patients who received pelvic RT showed that *Eubacterium eligens abundance* significantly decreased in the 5th week after RT compared with that at baseline. This species belongs to the family *Lachnospiraceae*, whose elevated abundance is significantly associated with fewer adverse effects in the GI tract.^33^ Aligned with this, patients without toxicity had a higher abundance of this species than those who experienced toxicity.

In addition to the abovementioned microbes, several other microbes showed dynamic trends in patients. Rather than focusing on a single microbe, microbial indices, such as the ratio of Firmicutes to Bacteroides, or dysbiosis indices, have been developed to conveniently represent gut dysbiosis in a group. For example, two studies^17^, ^28^ revealed that a high ratio of Firmicutes to Bacteroides was associated with RT‐induced diarrhea. Additionally, the dysbiosis index developed based on enriched and depleted taxa in Crohn's disease was used.^34^, ^35^ However, those indices did not show any significant relationship in the current study population. Instead, adopting the formulation of Nakatsu et al.,^22^ we reinvented the MCPI as an RT‐related microbial signature based on enriched microbes in patients who experienced toxicity compared to those who experienced no toxicity. The MCPI seemed to represent dynamics in the gut microbiome according to pelvic RT. Indeed, a significant difference in MCPI between patients with and without toxicity was found at 5 weeks after the start of RT.

In terms of predicted metabolic pathways, we found several differential functional pathways according to timepoints. Of note, poRT‐3 m seemed to show a different pattern compared to other timepoints. Except for poRT‐3 m, we identified a certain pattern in which the change induced by pelvic RT mostly recovered to the status of prRT in patients who experienced toxicity. For example, steroid hormone biosynthesis may be involved in anti‐inflammation for enteritis by RT. Additionally, the HIF‐1 signaling pathway may be relevant to the radioprotective effect given that HIF‐1 can attenuate or protect against RT‐induced GI inflammation.^14^, ^36^ Also, we observed the increasing trend of PI3K‐Akt signaling pathway in patients showing no toxicity. Inhibition of the PI3K‐Akt pathway has been shown to impair DNA repair after ionizing radiation in glioblastoma cells,^37^ indicating that the DNA repair system may be activated by this pathway. Cytokines such as HGF, IGF‐I, and IL‐6 can confer protection to cells against apoptosis induced by radiation through the PI3K‐Akt pathway.^38^ Thus, we speculate that activation of the pathway may lead to the mitigate or protect the bowel epithelial cells. These functional findings are hypothesis‐generating and should be validated in a whole metagenome sequencing study.

There are limitations in the current study. First, due to the small number of patients, there is the possibility of bias in conclusions regarding the relationship between a certain microbe and RT toxicity. Antihormone therapy, found to be used in 75 of the study population, has the potential to influence the composition of the gut microbiome,^39^ which can further modulate the gut microbial profile. The causal relationship between the two remains unclear. Nevertheless, microbiome studies in patients with prostate cancer are rare, and to our knowledge, five stool collections per patient have rarely been performed in previous studies. Second, the validity of an RT‐related MCPI should be examined in other cancers, and metabolic pathways changed during RT should be verified with metagenome sequencing.

## 5 CONCLUSION

In conclusion, we showed dysbiosis in the gut microbiome generated by RT in patients with prostate cancer who experienced RT toxicity. Reduced diversity and elevated RT‐related MCPI could be used for developing individualized RT. Longitudinal analysis revealed dynamic changes in several microbes and metabolic pathways, which should be validated in a whole metagenome sequencing study.

## AUTHOR CONTRIBUTIONS

**Bum‐Sup Jang:** Formal analysis (lead); writing – original draft (lead); writing – review and editing (lead). **Moon Gyu Chung:** Investigation (supporting); methodology (supporting); resources (supporting). **Dong Soo Lee:** Conceptualization (lead); data curation (lead); funding acquisition (lead); project administration (lead); writing – review and editing (lead).

## CONFLICT OF INTEREST STATEMENT

The authors declare no competing interests.

## ETHICS STATEMENT

This prospective cohort study was approved by the Catholic Medical Center Ethics Committee (approval No: UC19TESE0052).

## Supporting information


Figures S1–S2

Tables S1‐S2

## Data Availability

The data generated in this study are available within the article and its supplementary data files. Raw data are available upon request from the corresponding author. The SIMCAT package used in current study is available on GitHub repository (https://github.com/zellerlab/siamcat).

## References

[1] Ray GR , Cassady JR , Bagshaw MA . Definitive radiation therapy of carcinoma of the prostate. A report on 15 years of experience. Radiology. 1973;106:407‐418. 4630765 10.1148/106.2.407

[2] Zelefsky MJ , Fuks Z , Happersett L , et al. Clinical experience with intensity modulated radiation therapy (IMRT) in prostate cancer. Radiother Oncol. 2000;55:241‐249. 10869739 10.1016/s0167-8140(99)00100-0

[3] Jereczek‐Fossa BA , Orecchia R . Evidence‐based radiation oncology: definitive, adjuvant and salvage radiotherapy for non‐metastatic prostate cancer. Radiother Oncol. 2007;84:197‐215. 17532494 10.1016/j.radonc.2007.04.013

[4] Voet PW , Dirkx ML , Breedveld S , Al‐Mamgani A , Incrocci L , Heijmen BJ . Fully automated volumetric modulated arc therapy plan generation for prostate cancer patients. Int J Radiat Oncol Biol Phys. 2014;88:1175‐1179. 24529714 10.1016/j.ijrobp.2013.12.046

[5] Yu JB , Cramer LD , Herrin J , Soulos PR , Potosky AL , Gross CP . Stereotactic body radiation therapy versus intensity‐modulated radiation therapy for prostate cancer: comparison of toxicity. J Clin Oncol. 2014;32:1195‐1201. 24616315 10.1200/JCO.2013.53.8652 PMC3986382

[6] Mellon EA , Javedan K , Strom TJ , et al. A dosimetric comparison of volumetric modulated arc therapy with step‐and‐shoot intensity modulated radiation therapy for prostate cancer. Pract Radiat Oncol. 2015;5:11‐15. 25413432 10.1016/j.prro.2014.03.003

[7] Scobioala S , Kittel C , Elsayad K , et al. A treatment planning study comparing IMRT techniques and cyber knife for stereotactic body radiotherapy of low‐risk prostate carcinoma. Radiat Oncol. 2019;14:143. 31399115 10.1186/s13014-019-1353-6 PMC6689170

[8] Kang MJ , Won YJ , Lee JJ , et al. Cancer statistics in Korea: incidence, mortality, survival, and prevalence in 2019. Cancer Res Treat. 2022;54:330‐344. 35313102 10.4143/crt.2022.128 PMC9016309

[9] Matzinger O , Duclos F , van den Bergh A , et al. Acute toxicity of curative radiotherapy for intermediate‐ and high‐risk localised prostate cancer in the EORTC trial 22991. Eur J Cancer. 2009;45:2825‐2834. 19682889 10.1016/j.ejca.2009.07.009

[10] Katz AJ , Kang J . Quality of life and toxicity after SBRT for organ‐confined prostate cancer, a 7‐year study. Front Oncol. 2014;4:301. 25389521 10.3389/fonc.2014.00301 PMC4211385

[11] Schaake W , Wiegman EM , de Groot M , et al. The impact of gastrointestinal and genitourinary toxicity on health related quality of life among irradiated prostate cancer patients. Radiother Oncol. 2014;110:284‐290. 24411226 10.1016/j.radonc.2013.11.011

[12] Zelefsky MJ , Poon BY , Eastham J , Vickers A , Pei X , Scardino PT . Longitudinal assessment of quality of life after surgery, conformal brachytherapy, and intensity‐modulated radiation therapy for prostate cancer. Radiother Oncol. 2016;118:85‐91. 26780999 10.1016/j.radonc.2015.11.035 PMC4848377

[13] D'Avino V , Palma G , Liuzzi R , et al. Prediction of gastrointestinal toxicity after external beam radiotherapy for localized prostate cancer. Radiat Oncol. 2015;10:80. 25890376 10.1186/s13014-015-0389-5 PMC4404272

[14] Olcina MM , Giaccia AJ . Reducing radiation‐induced gastrointestinal toxicity‐—the role of the PHD/HIF axis. J Clin Invest. 2016;126:3708‐3715. 27548524 10.1172/JCI84432 PMC5096800

[15] Kinross JM , Darzi AW , Nicholson JK . Gut microbiome‐host interactions in health and disease. Genome Med. 2011;3:14. 21392406 10.1186/gm228 PMC3092099

[16] Shreiner AB , Kao JY , Young VB . The gut microbiome in health and in disease. Curr Opin Gastroenterol. 2015;31:69‐75. 25394236 10.1097/MOG.0000000000000139 PMC4290017

[17] Wang A , Ling Z , Yang Z , et al. Gut microbial dysbiosis may predict diarrhea and fatigue in patients undergoing pelvic cancer radiotherapy: a pilot study. PLoS One. 2015;10:e0126312. 25955845 10.1371/journal.pone.0126312 PMC4425680

[18] Wilson ID , Nicholson JK . Gut microbiome interactions with drug metabolism, efficacy, and toxicity. Transl Res. 2017;179:204‐222. 27591027 10.1016/j.trsl.2016.08.002 PMC5718288

[19] Oh B , Eade T , Lamoury G , et al. The gut microbiome and gastrointestinal toxicities in pelvic radiation therapy: a clinical review. Cancer. 2021;13:2353. 10.3390/cancers13102353 PMC8153110 34068216

[20] Lozupone C , Knight R . UniFrac: a new phylogenetic method for comparing microbial communities. Appl Environ Microbiol. 2005;71:8228‐8235. 16332807 10.1128/AEM.71.12.8228-8235.2005 PMC1317376

[21] Wirbel J , Zych K , Essex M , et al. Microbiome meta‐analysis and cross‐disease comparison enabled by the SIAMCAT machine learning toolbox. Genome Biol. 2021;22:93. 33785070 10.1186/s13059-021-02306-1 PMC8008609

[22] Nakatsu G , Li X , Zhou H , et al. Gut mucosal microbiome across stages of colorectal carcinogenesis. Nat Commun. 2015;6:8727. 26515465 10.1038/ncomms9727 PMC4640069

[23] Dou Y , Ma C , Wang K , et al. Dysbiotic tumor microbiota associates with head and neck squamous cell carcinoma outcomes. Oral Oncol. 2022;124:105657. 34915261 10.1016/j.oraloncology.2021.105657

[24] Langille MG , Zaneveld J , Caporaso JG , et al. Predictive functional profiling of microbial communities using 16S rRNA marker gene sequences. Nat Biotechnol. 2013;31:814‐821. 23975157 10.1038/nbt.2676 PMC3819121

[25] Ott SJ , Musfeldt M , Wenderoth DF , et al. Reduction in diversity of the colonic mucosa associated bacterial microflora in patients with active inflammatory bowel disease. Gut. 2004;53:685‐693. 15082587 10.1136/gut.2003.025403 PMC1774050

[26] Manichanh C , Rigottier‐Gois L , Bonnaud E , et al. Reduced diversity of faecal microbiota in Crohn's disease revealed by a metagenomic approach. Gut. 2006;55:205‐211. 16188921 10.1136/gut.2005.073817 PMC1856500

[27] Boland K , Bedrani L , Turpin W , et al. Persistent diarrhea in patients with Crohn's disease after mucosal healing is associated with lower diversity of the intestinal microbiome and increased dysbiosis. Clin Gastroenterol Hepatol. 2021;19:296‐304.e3. 32220613 10.1016/j.cgh.2020.03.044 PMC7511440

[28] Wang Z , Wang Q , Wang X , et al. Gut microbial dysbiosis is associated with development and progression of radiation enteritis during pelvic radiotherapy. J Cell Mol Med. 2019;23:3747‐3756. 30908851 10.1111/jcmm.14289 PMC6484301

[29] Mitra A , Grossman Biegert GW , Delgado AY , et al. Microbial diversity and composition is associated with patient‐reported toxicity during chemoradiation therapy for cervical cancer. Int J Radiat Oncol Biol Phys. 2020;107:163‐171. 31987960 10.1016/j.ijrobp.2019.12.040 PMC7932475

[30] Reis Ferreira M , Andreyev HJN , Mohammed K , et al. Microbiota‐ and radiotherapy‐induced gastrointestinal side‐effects (MARS) study: a large pilot study of the microbiome in acute and late‐radiation enteropathy. Clin Cancer Res. 2019;25:6487‐6500. 31345839 10.1158/1078-0432.CCR-19-0960

[31] Forbes JD , Chen CY , Knox NC , et al. A comparative study of the gut microbiota in immune‐mediated inflammatory diseases‐does a common dysbiosis exist? Microbiome. 2018;6:221. 30545401 10.1186/s40168-018-0603-4 PMC6292067

[32] Nam YD , Kim HJ , Seo JG , Kang SW , Bae JW . Impact of pelvic radiotherapy on gut microbiota of gynecological cancer patients revealed by massive pyrosequencing. PLoS One. 2013;8:e82659. 24367534 10.1371/journal.pone.0082659 PMC3867375

[33] Guo H , Chou WC , Lai Y , et al. Multi‐omics analyses of radiation survivors identify radioprotective microbes and metabolites. Science. 2020;370:eaay9097. 33122357 10.1126/science.aay9097 PMC7898465

[34] Gevers D , Kugathasan S , Denson LA , et al. The treatment‐naive microbiome in new‐onset Crohn's disease. Cell Host Microbe. 2014;15:382‐392. 24629344 10.1016/j.chom.2014.02.005 PMC4059512

[35] Wei S , Bahl MI , Baunwall SMD , Hvas CL , Licht TR . Determining gut microbial dysbiosis: a review of applied indexes for assessment of intestinal microbiota imbalances. Appl Environ Microbiol. 2021;87:e00395‐21. 33741632 10.1128/AEM.00395-21 PMC8208139

[36] Tian T , Zhao Y , Yang Y , et al. The protective role of short‐chain fatty acids acting as signal molecules in chemotherapy‐ or radiation‐induced intestinal inflammation. Am J Cancer Res. 2020;10:3508‐3531. 33294252 PMC7716145

[37] Li HF , Kim JS , Waldman T . Radiation‐induced Akt activation modulates radioresistance in human glioblastoma cells. Radiat Oncol. 2009;4:43. 19828040 10.1186/1748-717X-4-43 PMC2765447

[38] Zhan M , Han ZC . Phosphatidylinositide 3‐kinase/AKT in radiation responses. Histol Histopathol. 2004;19:915‐923. 15168354 10.14670/HH-19.915

[39] Fujita K , Matsushita M , De Velasco MA , et al. The gut‐prostate Axis: a new perspective of prostate cancer biology through the gut microbiome. Cancer. 2023;15:1375. 10.3390/cancers15051375 PMC10000196 36900168

