# Supplementary material for: Association between gut microbial change and acute gastrointestinal toxicity in patients with prostate cancer receiving definitive radiation therapy
Source: Cancer Med. 2023 Nov 3;12(22):20727–35. doi: 10.1002/cam4.6636 (PMC10709749; doi:10.1002/cam4.6636)
Supplement: Supplementary file 1 — Figures S1–S2 [file CAM4-12-20727-s001.zip › Supplementary Figure captions.docx]

FIGURE S1. PERMANOVA according to sampling time stratified by the presence of toxicity.

FIGURE S2. All differential taxa according to the presence of toxicity at the family level (A), genus level (B), and species level (C).
