# Supplementary figures and images for: Association between gut microbial change and acute gastrointestinal toxicity in patients with prostate cancer receiving definitive radiation therapy
Source: Cancer Med. 2023 Nov 3;12(22):20727–35. doi: 10.1002/cam4.6636 (PMC10709749; doi:10.1002/cam4.6636)

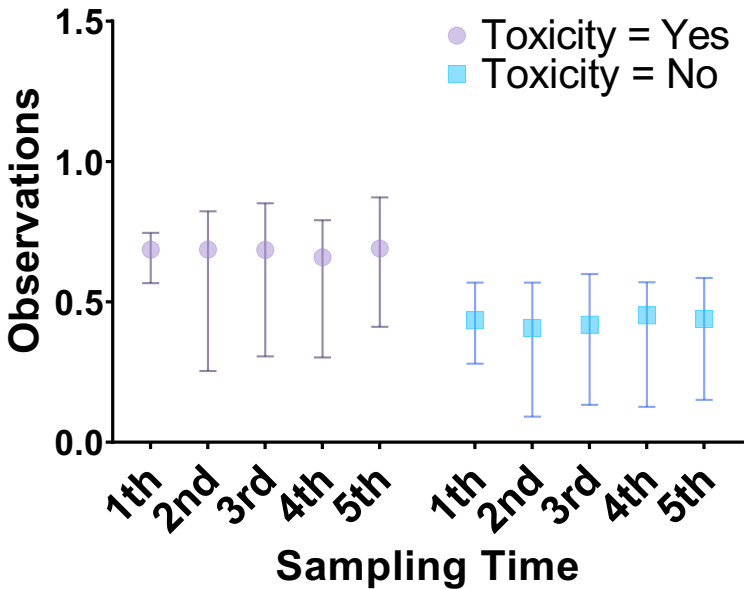

Supplement: Supplementary file 1 — Figures S1–S2 [file CAM4-12-20727-s001.zip › cam46636-sup-0001-Supp Fig 1.pdf]

A. Family Level

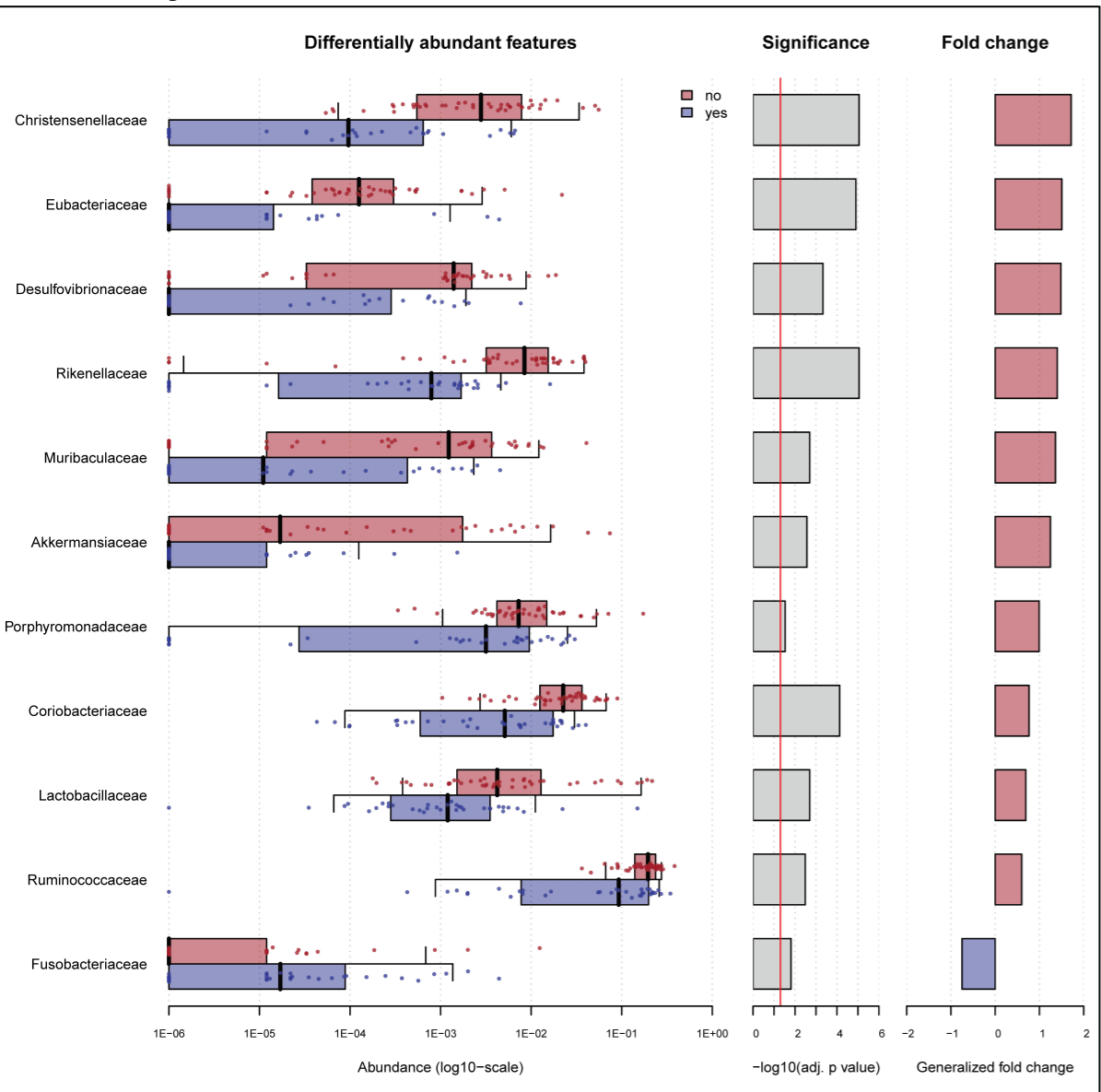

B. Genus Level

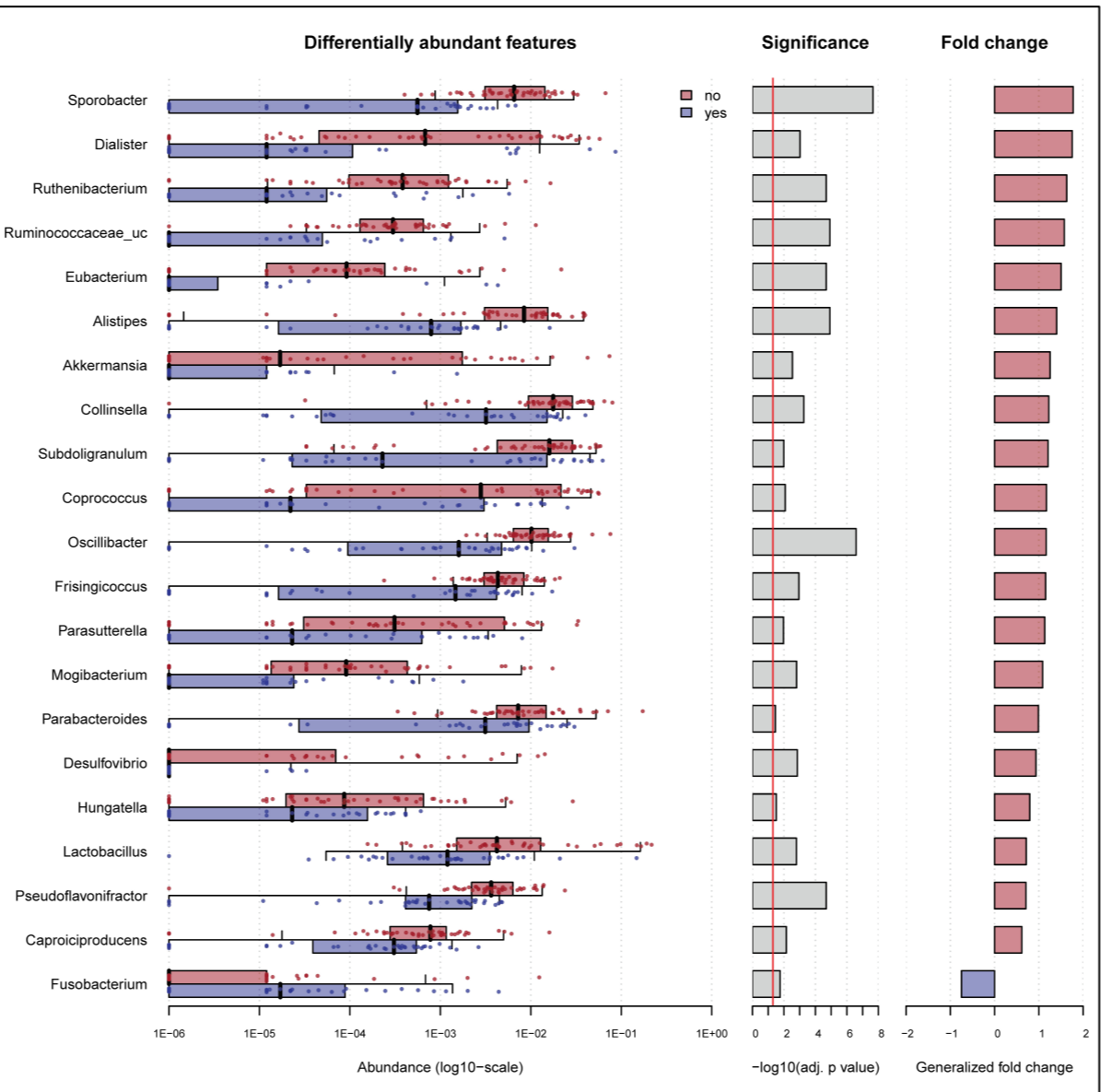

C. Species Level

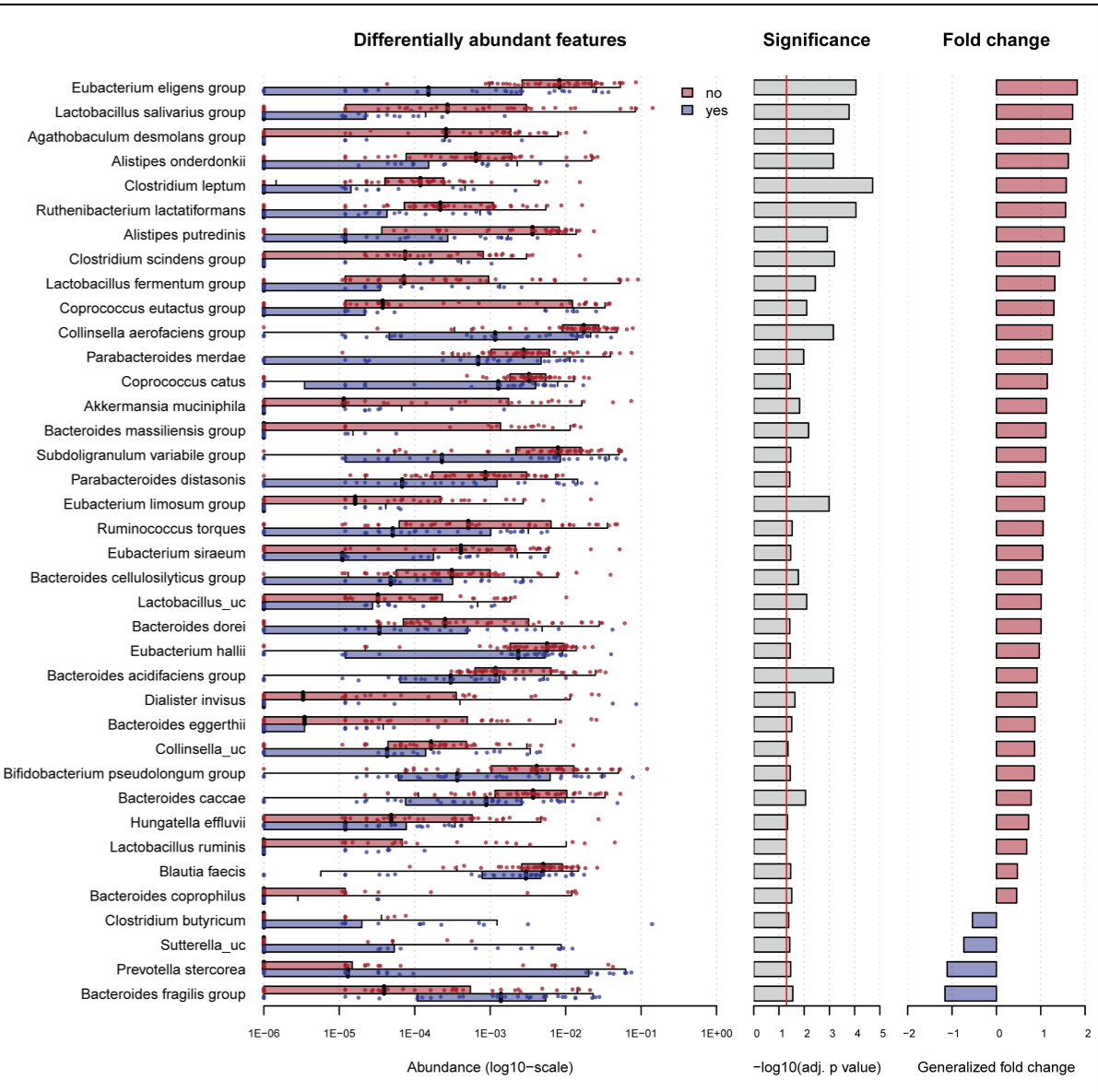

Supplement: Supplementary file 1 — Figures S1–S2 [file CAM4-12-20727-s001.zip › cam46636-sup-0002-Supp Fig 2.pdf]
