# Supplementary material for: Association between gut microbial change and acute gastrointestinal toxicity in patients with prostate cancer receiving definitive radiation therapy
Source: Cancer Med. 2023 Nov 3;12(22):20727–35. doi: 10.1002/cam4.6636 (PMC10709749; doi:10.1002/cam4.6636)
Supplement: Supplementary file 2 — Tables S1‐S2 [file CAM4-12-20727-s002.docx]

**Supplementary Table 1. The number of patients representing toxicity or no toxicity in each time point**

|  | **Timepoints (After RT)** | | | | | |
| --- | --- | --- | --- | --- | --- | --- |
|  | **1-week** | **2-week** | **3-week** | **4-week** | **5-week** | **6-week** |
| **Toxicity (N)** | **0** | **2** | **1** | **4** | **0** | **0** |
| **No toxicity (N)** | **16** | **14** | **15** | **12** | **16** | **16** |

**Supplementary Table 2. Predicted pathway abundances according to timepoints, stratified by toxicity**

| Pathway | Definition | Predicted Abundances | | | | | P |  |
| --- | --- | --- | --- | --- | --- | --- | --- | --- |
| **Toxicity = Y** | | prRT | RT-2w | RT-5w | poRT-1m | poRT-3m |  | Trend |
| ko00720 | Carbon fixation pathways in prokaryotes | 0.681 | 0.679 | 0.673 | 0.672 | 0.614 | 0.038 | Decreasing |
| ko03070 | Bacterial secretion system | 0.570 | 0.564 | 0.549 | 0.546 | 0.508 | 0.019 | Decreasing |
| ko00471 | D-Glutamine and D-glutamate metabolism | 0.375 | 0.419 | 0.242 | 0.327 | 0.263 | 0.048 | Increasing-decreasing |
| ko00140 | Steroid hormone biosynthesis | 0.324 | 0.388 | 0.262 | 0.310 | 0.156 | 0.038 | Increasing-decreasing |
| ko05133 | Pertussis | 0.210 | 0.221 | 0.187 | 0.205 | 0.153 | 0.015 | Increasing-decreasing |
| ko04923 | Regulation of lipolysis in adipocytes | 0.000 | 0.010 | 0.004 | 0.004 | 0.014 | 0.040 | Increasing-decreasing |
| **Toxicity = N** | |  |  |  |  |  |  |  |
| ko04151 | PI3K-Akt signaling pathway | 0.220 | 0.244 | 0.269 | 0.280 | 0.281 | 0.047 | Increasing |

Abbreviations: RT, radiation therapy; prRT, pre-RT; RT-2w, 2 weeks after the start of RT; RT-5w, 5 weeks after the start of RT; poRT-1m, 1 month later from the end of RT; poRT-3m, 3 months later from the end of RT.
